# Engineering recombinantly expressed lectin-based antiviral agents

**DOI:** 10.3389/fcimb.2022.990875

**Published:** 2022-09-23

**Authors:** Irene Maier

**Affiliations:** Department of Environmental Health Sciences, University of California Los Angeles, CA, Los Angeles, United States

**Keywords:** lectin, cyanovirin-N, carbohydrate-binding agent, virus, SARS-CoV-2

## Abstract

Cyanovirin-N (CV-N), a lectin from *Nostoc ellipsosporum* was found an infusion inhibitory protein for human immunodeficiency virus (HIV)-1. A tandem-repeat of the engineered domain-swapped dimer bound specific sites at hemagglutinin (HA), Ebola and HIV spike glycoproteins as well as dimannosylated HA peptide, N-acetyl-D-glucosamine and high-mannose containing oligosaccharides. Among these, CV-N bound the severe acute respiratory syndrome coronavirus-2 (SARS-CoV-2) spike protein at a dissociation constant (K_D_) of 18.6 µM (and K_D_=260 µM to RBD), which was low-affinity carbohydrate-binding as compared with the recognition of the other viral spikes. Binding of dimannosylated peptide to homo-dimeric CVN2 and variants of CVN2 that were pairing Glu-Arg residues sterically located close to its high-affinity carbohydrate binding sites, was measured using surface plasmon resonance (SPR) and isothermal titration calorimetry (ITC). Binding affinity increased with polar interactions, when the mutated residues were used to substitute a single, or two disulfide bonds, in CVN2. Site-specific N-linked glycans on spikes were mediating the infection with influenza virus by broadly neutralizing antibodies to HA and lectin binding to HA was further investigated *via* modes of saturation transfer difference (STD)-NMR. Our findings showed that stoichiometry and the lectin’s binding affinity were revealed by an interaction of CVN2 with dimannose units and either the high- or low-affinity binding site. To understand how these binding mechanisms add to viral membrane fusion we compare our tested HA-derived peptides in affinity with SARS-CoV-2 glycoprotein and review lectins and their mechanisms of binding to enveloped viruses for a potential use to simulate neutralization ability.

## Introduction

Cyanobacterial lectin CV-N displayed antiviral activity, which is mediated by nanomolar binding to high-mannose oligosaccharide modifications on envelope spike proteins (Mazur-Marzec et al., 2021), against human immunodeficiency virus 1 (HIV-1) (Boyd, 1997; Bolmstedt et al., 2001), influenza (O’Keefe et al., 2003), herpes virus, hepatitis C, severe acute respiratory syndrome coronavirus (SARS-CoV)-2 (Naidoo et al., 2021; Li et al., 2022), among others, and binds Ebola virus (Jensen et al., 2014). Structural analyses and binding affinity assays indicated cross-linking of two high- or low-affinity carbohydrate binding sites in a domain-swapped CVN2 dimer (Boyd, 1997; Bewley et al., 2002; Barrientos et al., 2006) by bivalent binding in the micromolar range to enhance avidity to viral envelope glycoproteins (Schilling et al., 2020) and to inhibit viral entry (Boyd, 1997; O’Keefe et al., 2003; Barrientos et al., 2006). Selective binding of the terminal-accessible disaccharide Manα1-2Manα on oligomannose-8 D1D3 arms and oligomannose-9 was comprised by two binding sites of differing affinities located on opposite protein protomers, thereby reaching nanomolar binding affinities (Bewley, 2001; Bewley and Otero-Quintero, 2001). Thus, CVN2 is considered a pseudo-antibody concerning its application to bind epitopes on HIV gp120 similar to virus-neutralizing antibodies (Shenoy et al., 2002; Keeffe et al., 2011).

## Lectins from natural sources function as viral entry inhibitors

A class of cyanometabolites that exhibit antiviral effects were focused on lectins and polysaccharides (Mazur-Marzec et al., 2021), and specifically Griffithsin (GRFT), a red-alga-derived lectin showed broad binding to enveloped viruses in combination with sulfated polysaccharides against SARS-CoV-1 and 2 (Alam et al., 2021; Alsaidi et al., 2021). The use of other carbohydrate-binding agents, such as lectins from plants, fungi, and prokaryotes (Gupta et al., 2020), or anti-HIV antibiotics Pradimicin A (Tanabe-Tochikura et al., 1990) was limited due to unfavorable responses like immunogenicity, mitogenicity, hemagglutination, inflammatory activity, and cellular toxicity. Most of them showed antiviral activities against coronavirus though (Gupta et al., 2020). Wheat germ agglutinin (WGA) and lentil lectin were evaluated for antiviral efficacy through direct binding to SARS-CoV-2 Spike (S) protein (Auth et al., 2021; Wang et al., 2021) and neutralization activity against SARS-CoV-2 and its major variants of concern (Alpha, Beta) (Auth et al., 2021), and lentil lectin blocked binding to angiotensin-converting enzyme 2 (ACE2) receptor (Mazur-Marzec et al., 2021; Wang et al., 2021). The *Lens culinaris*-derived lentil lectin showed broader activity, weak hemagglutination activity at 1 mg/mL and no cytotoxicity activity, and no weight loss was found in the single injection mouse experiment (Wang et al., 2021). To systematically identify lectins that bind to the trimeric S protein and receptor-binding domain (RBD) of SARS-CoV-2, researchers searched for all annotated carbohydrate recognition domains (CRDs) of mouse C-type lectins, galectins and sialic acid-binding immunoglobulin-type lectins (Siglecs). Of 168 annotated CRDs, 143 lectin-CRDs were expressed as IgG2a-Fc fusion proteins from human HEK293F cells and two lectins, Clec4g and CD209c, were then selected (Hoffmann et al., 2021) that, like DC/L-SIGN, were evaluated to bind S protein of SARS-CoV-2 at the RBD with ACE2 interfering interaction or sterically blocking the receptor binding (Lempp et al., 2021; Thépaut et al., 2021).

## Structural features for targeting glycoproteins

Surface-expressed membrane glycoproteins hemagglutinin (HA) and neuraminidase (NA) on influenza A virus are known for tetherin antagonism in a strain-specific manner (Gnirss et al., 2015), as they facilitate recognition of host receptor binding sites (Knossow and Skehel, 2006; Schmidt et al., 2015). HA binding is reported for broadly neutralizing antibodies, lectins, and also specific antibodies with highly variant antigenic sites (Knossow and Skehel, 2006; Schmidt et al., 2015; Wu and Wilson, 2017; Maier et al., 2021), but conserved epitopes on the homotrimeric membrane glycoprotein at the globular head domain (Bizebard et al., 1995; Fleury et al., 1998; Fleury et al., 1999; Krause et al., 2011; Benjamin et al., 2014; Schmidt et al., 2015; Nogales et al., 2018; Qiu et al., 2020) and stem region (Ekiert et al., 2009; Corti et al., 2011; Ekiert et al., 2011; Lee et al., 2012; Nachbagauer et al., 2014; Chen et al., 2021).

Current vaccines are effective but strain specific due to their focus on the immunodominant globular head domain of the HA (Krause et al., 2011; Schmidt et al., 2015; Raymond et al., 2018; Liao et al., 2020; Qiu et al., 2020). Contrarily, non-neutralizing antibodies destabilized the HA stem region, resulting in antibody-dependent enhancement of influenza disease, and enhanced virus fusion kinetics and manifestation of the respiratory disease in preclinical studies by treatment with two monoclonal antibodies (mAbs) following H3N2 viral challenge (Winarski et al., 2019). Structural features allowed mapping of single amino-acid mutations on HA (HA1 and HA2; where both are linked by disulfide bridges) that increase resistance to broad antibodies to H1 strains and show escape from antibody neutralization. Those antibodies targeting the H1 HA stalk [FI6v3 (Corti et al., 2011) and C179 (Dreyfus et al., 2013)] were broader, harder to escape, but less mutationally tolerating than other antibodies which targeted the head domain (Doud et al., 2018; Chen et al., 2021). Several studies showed evidence that it is possible to select antigenic mutants with broad antibodies, demonstrating that these epitopes are not entirely resistant to change (Doud et al., 2018; Wu et al., 2020a). More stem-specific antibodies were directed against a chimeric mono-glycosylated HA vaccine comprised of consensus sequences of avian H5 and H1 strains. The monosaccharide N-acetylglucosamine (GlcNAc) at the HA glycosite was attributed to better neutralization and cross-protection against H1, H3, H5, and H7 strains and subtypes, and overall vaccine efficacy was increased when the recombinant HA antigen-based vaccine was combined with a glycolipid adjuvant (Liao et al., 2020). Interestingly, N-linked glycans on the H5 antigen globular head domain and glycan-unmasking at the stem region elicit broad neutralizing antibodies to cross-protect against various H5N1 clades of virus infection (Chen et al., 2021); while the mechanism of introducing additional N-glycosylation sites was recently also applied to the modification of SARS-CoV-2 S glycoprotein (Galili, 2020; Lin et al., 2021). Glycans found on the SARS-CoV-2 S trimer (Watanabe et al., 2020) revealed few, but invariant interactions with human neutralizing antibodies to SARS-CoV-2 WT, the Alpha, Delta, Lambda, and Omicron variants (Wang et al., 2022), from which many were isolated, investigated during the current pandemics (Robbiani et al., 2020; Barnes et al., 2020a) and classified according to their binding capacities for epitopes on S trimer. The related “up” conformations of RBD (Lv et al., 2020; Pinto et al., 2020; Wu et al., 2020b), and down conformation of the RBD of the closed, prefusion S trimer (Zost et al., 2020a; Barnes et al., 2020b) were addressed. Around 2191 structures of SARS-CoV-2 S protein were published in the protein data bank until January 2022 and glycosylation varied according to the method of analyses used for structural classification of correlates, as well as immune pressure (Cao et al., 2020; Ju et al., 2020; Kreer et al., 2020; Shi et al., 2020; Walls et al., 2020a; Walls et al., 2020b; Wu et al., 2020c; Yu et al., 2020; Zost et al., 2020b). A comparison of CV-N binding to HA, HA top and RBD on human 2019-nCoV (Wuhan-Hu-1-2019 novel coronavirus) was examined (Maier, 2022). Using cryo-electron microscopy and binding assays, Pinto and coworkers described a mAb S309 and a class 3 mAb, that potentially recognized an epitope and glycan (N343 on SARS-CoV-2) that was found to be conserved within *Sarbecovirus subgenus*, without competing with receptor attachment (Pinto et al., 2020). The deletion of the glycosylation site at N165 on S protein by mutation N165A induced up-state to RBD. The solvent released N234-glycans also had direct contacts with the up-RBD in the WT and G614 S trimers, the state which is favored upon binding to ACE2 (Wang et al., 2022).

## Mechanisms of cyanovirin-N binding to viral spikes

Influenza A antigen binding (H3N2) (Benjamin et al., 2014; Zost et al., 2017; Qiu et al., 2020) to one high-affinity binding site in CV-N, or two high-affinity binding sites in covalently linked dimeric CVN2 was determined to have equilibrium dissociation constants (K_D_)=5.7 nM and K_D_=2.7 nM, respectively. Selected immunoglobulin classes addressed specific and invariant structural patterns, however, which provided a substrate for affinity maturation in the membrane-anchored HA regions (Lingwood et al., 2012; Otterstrom et al., 2014). Our knowledge is incomplete on the variety and localization of epitopes on the stem of HA1 and HA2 that both involve epitope structures for glycan-targeting by highly neutralizing antibodies as compared with lectin binding (Ekiert et al., 2009).

To investigate the binding of homogenously glycosylated targets on HA, we chemically synthesized di- and tri-mannose moieties *via* azido glycosylation (Salunke et al., 2011), therefore facilitating site-specific interactions with CV-N derivatives. Whereas the glycan shield on the membrane-distal HA top part induced high-affinity binding to CV-N, CVN2 binding to HA close to a stabilizing disulfide bridge of HA top [4 N-linked glycans at N54, N97, N181, N301 (Bizebard et al., 1995)] has further been observed at its low-affinity sites. HA binding to at least one high-affinity carbohydrate binding site (H) of CVN2 has been examined at K_D_ of 275 nM (A/Wisconsin/67/05) (Schilling et al., 2020), and Ebola GP1,2 bound to 2H of CVN2 with affinities in the lower nanomolar range (K_D_=26 nM) as measured *via* surface plasmon resonance (SPR) (Maier et al., 2021). Higher density of glycans on HA protein achieved binding with polar Glu-Arg residues instead of cystines in CVN2L0, and an association between respective mannosylated peptides (K_D_=10 µM for dimannosylated HA peptide, DM) and CVN2L0 or mutated Glu-Arg pairing near the HA binding pockets (Schilling et al., 2020). Testing H on domains B, which was impacted by replacing a disulfide bond into ionic residues, and domain A forming the low-affinity carbohydrate binding site (L), the dimeric domain-swapped CVN2L0 molecule showed binding to HA top region and the whole molecule. Binding affinity was presented as SPR K_D_ values: A variant with altered binding-affinity was expressed in *E. coli*, purified, and positively tested for binding to HA-protein (H3N2) and DM, and showed a conformational change upon binding HA with either H or L carbohydrate binding sites and K_D1_= 49 nM and K_D2_ = 8 µM (Schilling et al., 2020; Maier et al., 2021). Disulfide bond variants were created by substitution of Cys and insertion of polar residue pairs Glu – Arg with slightly decreasing thermal stability and helical structures in V4 and V5. As the number of disulfide bonds in vicinity to the glycan-targeting pocket in CVN2 decreased from 4 (CVN2L0) to 2 (variants V3-5), binding affinity to HA protein decreased (Schilling et al., 2020).

Next, we were also interested in investigating the potential binding of CV-N to SARS-CoV-2 spikes *via* its RBD and CVN2 to RBD, assuming specific targeting of possibly conserved carbohydrates on RBD S1 subunit (Pinto et al., 2020; Barnes et al., 2020a). SARS-CoV-2 glycosylation of S trimer was characterized by 14 N-linked complex, 6 N-linked hybrid-type, 2 high-mannose glycosylation sites, and O-linked serine-attached and O-linked threonine glycosylation sites (Pinto et al., 2020; Watanabe et al., 2020). The monomeric mutant CVN-E41A was suspected to destabilize pseudo-domain B or to interrupt connectivity with the second domain A (Maier, 2022), such as found in CV-N WT (Yang et al., 1999). CVN-E41A monomer revealed enhanced protein stability upon binding to S protein, although this mutation site abolished a contacting residue between H and L, and reduced strength of molecular binding to GlcNAc. Binding of CVN-E41A to SARS-CoV-2 S protein, bearing complex-type N-linked glycosylation and O-glycosylation, was achieved in the SPR at micromolar concentrations (Maier, 2022) through the rather small number of high-mannose containing oligosaccharides *via* SARS-CoV-2 RBD (Watanabe et al., 2020; Wang et al., 2022). CV-N WT binding to SARS-CoV-2 RBD was measured at weaker affinity (K_D_=260 µM) (Maier, 2022) as compared with binding of CV-N to HA (A/New York/55/04, K_D_=5.7 nM) (Maier et al., 2021) and binding to S protein [K_D_=18.6 µM, (Maier, 2022)]. In comparison, the biologically relevant human ACE2 interaction with the SARS-CoV-2 RBD was reported at K_D_=4.7 nM (Lan et al., 2020).

## Discussion

Antiviral lectins showed neutralization ability alike broadly neutralizing antibodies to be restored by dimerization and to require two H, whereas a single binding site to HIV spike glycoproteins alone was not sufficient to reveal neutralization of enveloped viruses itself (Keeffe et al., 2011). These interactions were verified by generating knock-out variants in the binding site to correlate binding affinities to *in silico* predicted glycosylation (Maier et al., 2021). Thus, we aim to compare our previously tested chemically mannosylated HA peptides in binding affinity and specificity with short peptide sequences from SARS-related 2019 novel coronavirus (nCoV) spikes and SARS-CoV-2, which are naturally modified by different N-linked glycosylation sites (Kumar et al., 2020; Watanabe et al., 2020) and O-linked glycosylation (Watanabe et al., 2020, **Table 1**).

**Table 1:** Literature on viral spike glycoproteins GP120, HA, and SARS-CoV (2) variants.

Cited Literature	Ref. in Manuscript	Antigenic Target (to Lectin)
Schilling, P.E., et al., 2020	10	HA bound by CVN2
Bewley, C.A., and Otero-Quintero, S. 2001	11	GP120 bound by CV-N
Bewley, C.A. 2001	12	GP120 bound by CV-N
Shenoy, S.R., et al. 2002	13	GP120 bound by CV-N
Keeffe, J.R., et al., 2011	14	GP120 bound by CVN2
Alsaidi, S., et al., 2021	15	SARS-CoV-1 and -2 bound by Griffithsin
Auth, J., et al., 2021	19	SARS-CoV-2 bound by lectin from *Triticum vulgaris* (WGA)
Wang, W., et al., 2021	20	SARS-CoV-2 bound by lentil lectin from *Lens culinaris*
Hoffmann, D., et al., 2021	21	SARS-CoV-2
Thépaut, M., et al., 2021	22	SARS-CoV-2
Lempp, F.A., et al., 2021	23	SARS-CoV-2
Maier, I., et al., 2021	27	HA (Influenza A H3N2) bound by CV-N
Chen, T.H., et al., 2021	41	HA (Avian Influenza H5N1)
Liao, H.Y., et al., 2020	42	HA
Raymond, D.D., et al., 2018	43	HA
Winarski, K.L., et al., 2019	44	HA
Lin, W.S., et al., 2021	48	SARS-CoV-2
Galili, U. 2020	50	SARS-CoV-2
Watanabe, Y., et al. 2020	51	SARS-CoV-2
Wang, X., et al., 2022	52	SARS-CoV-2
Robbiani, D.F., et al., 2020	53	SARS-CoV-2
Barnes, C.O., et al., 2020	54	SARS-CoV-2
Lv, Z., et al., 2020	55	SARS-CoV, SARS-CoV-2
Pinto, D., et al., 2020	56	SARS-CoV, SARS-CoV-2
Wu, N.C., et al., 2020	57	SARS-CoV-2
Zost, S.J., et al., 2020	58	SARS-CoV-2
Barnes, C.O., et al., 2020	59	SARS-CoV-2
Ju, B., et al., 2020	60	SARS-CoV-2
Yu, F., et al., 2020	61	SARS-CoV, SARS-CoV-2
Wu, N.C., et al., 2020	62	SARS-CoV, SARS-CoV-2
Walls, A.C., et al., 2020a	63	SARS-CoV-2
Walls, A.C., et al., 2020b	64	SARS-CoV-2
Cao, Y., et al., 2020	65	SARS-CoV-2
Kreer, C., et al., 2020	66	SARS-CoV-2
Shi, R., et al., 2020	67	SARS-CoV-2
Zost, S.J., et al., 2020	68	SARS-CoV-2
Maier, I., 2022	69	HA (H3N2), SARS-CoV-2
Ekiert, D.C., et al. 2009	73	HA
Lan, J., et al. 2020	76	SARS-CoV, SARS-CoV-2

Binding to oligo-mannosides was usually measured by isothermal titration calorimetry (ITC) (Shenoy et al., 2002), and has been applied to CV-N, or mannose-dependent HIV-1 neutralizing 2G12, which bind overlapping epitopes on gp120 involving position 234 and the conserved N-linked GlcNAc at position N295 (Alexandre et al., 2010). More high-mannose glycans attached to N332 were recognized by 2G12 (Scanlan et al., 2002). Mannosylated peptides developed in this study were used as protein scaffolds for screening binding characteristics of antiviral agents by SPR and NMR. Saturation transfer difference (STD)-NMR spectroscopy allowed for characterization of carbohydrate binding by conformational selection (Angulo et al., 2010; Goldflam et al., 2012; Schilling et al., 2020). The STD effect was assigned to the alkyl side chain of T83 in the HA mono- or dimannosylated HA peptide (Schilling et al., 2020). The replacement of two disulfide bonds by exchange of cystines into Trp-Met pairs (forming CVN2L0-V3) may also be utilized to produce chemically induced dynamic nuclear polarization (CIDNP) signals after laser irradiation in the presence of a suitable radical pair-generating dye (Siebert et al., 1997; Schilling et al., 2020). Until today, this technique focused on a series of GlcNAc-binding plant lectins of increasing structural complexity (hevein, pseudohevein, Urtica dioica agglutinin and WGA), for which supporting structural NMR- or X-ray crystallography data was available (Siebert et al., 1997). Taken together, exposed N-linked glycans allowed interactions with, for example, C-type lectin receptor DC-SIGN through many viruses, such as SARS-CoV-2, Ebola, and HIV, and contributed to virus internalization and dissemination. In the context of the recent SARS-CoV-2 pandemic, involvement of DC-SIGN has been linked to severe cases of COVID-19 (Thépaut et al., 2021), possibly competing with neutralizing antibodies to the RBD-motif (Lempp et al., 2021). Binding of CVN-E41A to SARS-CoV-2 S protein, on the other hand, was without physiological relevance, but may trigger neutralization of coronavirus by antibodies targeting non-overlapping epitopes at the conserved site of RBD on S protein.

## Author contributions

The author designed and pursued the study, and described the work for the present manuscript.

## Conflict of interest

The author declares that the research was conducted in the absence of any commercial or financial relationships that could be construed as a potential conflict of interest.

## Publisher’s note

All claims expressed in this article are solely those of the authors and do not necessarily represent those of their affiliated organizations, or those of the publisher, the editors and the reviewers. Any product that may be evaluated in this article, or claim that may be made by its manufacturer, is not guaranteed or endorsed by the publisher.
